# Prevalence of Bullying and Its Association With Health-Related Quality of Life Among Adolescents in Jazan: A Cross-Sectional Study

**DOI:** 10.7759/cureus.28522

**Published:** 2022-08-29

**Authors:** Mona Elmahdy, Nada A Maashi, Shouq O Hakami, Maha A Fathi, Hanen I Alsuri, Shumukh H Hezymi, Ibrahim M Dighriri, Sarah Elrefai, Zenat Khired, Amani O Abdelmola

**Affiliations:** 1 Department of Family and Community Medicine, Jazan University, Jazan, SAU; 2 Department of Medicine, Jazan University, Jazan, SAU; 3 Department of Pharmacy, King Abdulaziz Specialist Hospital, Taif, SAU; 4 Department of Forensic Medicine and Toxicology, Faculty of Medicine, Jazan University, Jazan, SAU; 5 Department of Surgery, Jazan University, Jazan, SAU

**Keywords:** hrqol, jazan, adolescents, victim, bullying

## Abstract

Background and aim: Bullying is one of the most significant problems that has emerged across the world. It has negative effects on physical, psychological, and social health, especially among adolescents. No previous studies have been conducted in the Jazan region of Saudi Arabia to investigate the association between bullying and health-related quality of life (HRQOL). The study aimed to estimate the prevalence of bullying and its association with HRQOL among adolescents in Jazan.

Methods: A cross-sectional study was conducted among 461 adolescents in the Jazan region, Saudi Arabia. They were selected from nine schools by a convenient type of sampling, using snowball technique. HRQOL was tested using the Arabic version of KIDSCREEN-27. Data were collected using a web survey and analyzed by the Statistical Package for Social Sciences (SPSS) version 24 (Armonk, NY: IBM Corp.).

Results: The study revealed that 35.3% of all participants were labeled as victims. Victimization was more common among boys (17.78%) than girls (17.57%) (P = 0.001). Most of the victims were bullied verbally (75.6%), and most of them were bullied by friends (57.67%). Regarding sex differences, cyberbullying was more prevalent among girls (18.04%) than among boys (9.82%) (P = 0.01). Bullying was more common in elementary schools (39.5%) than in others. Associations were found between bullying and all aspects of HRQOL that were evaluated (P<0.0001).

Conclusions: Bullying prevalence is high among adolescents of the Jazan region and is associated with a poorer quality of life. This requires more attention from families and sectors of education, health, and social services.

## Introduction

Bullying is one of the most serious problems that has become widespread in modern societies. It happens everywhere, such as at home, school, or social events. It is defined as a form of aggressive behavior that aims to harm or cause inconvenience to another person [1]. Bullying research began in Europe in the 1970s, directed by Olweus in 1993, now considered the best international researcher on the issue [2]. However, he reported three characteristics that distinguish bullying from other forms of violence: intentionality, long-term repetition, and a power imbalance between bullies and victims [3]. Bullying can take various forms, including physical bullying (e.g., striking, kicking, tripping, and pinching), verbal bullying (e.g., name-calling, insults, and humiliation), social bullying (e.g., jokes to shame, ostracizing, spreading stories, inflicting harm to someone's social reputation), and cyberbullying (e.g., causing others distress by using cell phones and the internet) [4].

Several studies highlight the prevalence of bullying in different ways. The study by Craig et al. explored bullying based on data from 40 countries. The study indicated that the number of boys ranged from 8.6% to 45.2%, while the proportion of girls ranged from 4.8% to 35.8% [5]. Another study conducted in Mexico reveals that the prevalence varied across academic level, with 52.7% in elementary school and 28% in university [6]. The first nationally representative sample of adolescents in the Kingdom of Saudi Arabia (KSA) has recently revealed a prevalence of 25.0% bullying and 20.8% physical violence in school [7].

Research has shown that victimization is associated with negative physical and emotional health outcomes and social developmental negative consequences, as well as negative effects on life prospects, including academic achievement [8]. Furthermore, according to a previous study conducted by Wilkins-Shurmer et al., school bullying is a significant determinant of children's quality of life (QOL), and usually has negative long-term health implications [9].

The concept of quality of life is defined by the World Health Organization (WHO) as “an individual’s perception of their position in life in the context of the culture and value system in which they live and in relation to their goals, expectations, standards and concerns” [10]. The broad concept of health-related quality of life measures (HRQOL) provides researchers and practitioners with knowledge about the effects of a health condition or the impact of different interventions on various elements of HRQOL, as well as a framework for developing HRQOL-promoting techniques. However, in this study, we used KIDSCREEN-27 as a generic HRQOL measure for children and adolescents, which is a shortened version of KIDSCREEN-52, a test that has good psychometric qualities [11].

We hypothesize that bullying is negatively associated with HRQOL among adolescents in the Jazan region. Most previous studies in Saudi Arabia mainly focus on school-based bullying and its negative consequences. Also, inadequate studies have examined its association with HRQOL. To our knowledge, no previous studies relevant to this topic have been conducted in the Jazan region. Therefore, our objective was to estimate the prevalence of bullying, as well as its association with HRQOL, among adolescents in this region.

## Materials and methods

Study design and population

A cross-sectional study was conducted among adolescents living in the Jazan region of Saudi Arabia. Utilizing the snowball method, they were conveniently selected. It was conducted from August 2021 to March 2022 through a web-based survey. According to the official population census conducted in Jazan in 2016, a total of approximately 256,576 adolescents were registered. Males with made up 132,745, while females made up 123,831 [12]. We selected adolescents from 12 to 18 years of age. Because adolescence is a transitional phase in which many physical and psychological changes occur, we would like to see the impact of bullying on the quality of life during this period. We included those who agreed to participate and excluded those who refused to participate or were diagnosed with a psychological disease.

Procedures

Primary data collection was carried out using an online survey. Nine schools were selected conveniently from a list of all schools in two educational administrations in the Jazan region. In turn, the schools used social networks to send the questionnaire to parents and obtain their consent. Because our study interest comprises adolescents, we asked for parents' consent to distribute the questionnaire to their adolescent children, whether they were in the same school or not, or were illiterate.

Measurement

We used a three-part self-administered, semi-structured questionnaire. It was designed according to our research objectives and hypotheses, as well as to suit our culture using the Arabic language. The first part recorded sociodemographic details for gender, age, educational level, cohabitation status, and marital status. The second part examined bullying. An explanation of what is meant by "bullying" was included using Olweus' definition at the beginning of the section on bullying [13]. Then we used four questions to ask about bullying, three of them were to determine whether or not the adolescent had been bullied in the previous three months, as well as the source and type of bullying. The remaining question was one of the two global questions from the Olweus Bully/Victim Questionnaire that have previously been utilized in research [14]. This question asked how many times the adolescent bullied others in the previous three months. A five-point scale was used to answer them, where 1 shows “never”, 2 “only once or twice”, 3 “two or three times a month”, 4 “about once a week”, and 5 “several times a week”. The first two responses were recorded as “victims only," while the rest were "bully victims."

The third part of the questionnaire assessed the HRQOL using the Arabic version of KIDSCREEN-27. After obtaining approval from the KIDSCREEN group [11]. It contains 27 items distributed across five dimensions. Physical health (five items), psychological health (seven items), autonomy and parents (seven items), peers and social support (four items), and school and environment (four items). All responses are given on a five-point Likert scale, 1 = "not at all," 2 = "a little," 3 = "moderately," 4 = "much," and 5 = "very much". When scoring the questionnaire, four negatively worded items were reversed. T scores scaled with a mean of 50 and a standard deviation of 10 are calculated for each dimension using a scoring algorithm, while the overall KIDSCREEN score is calculated by adding all item responses. A higher HRQOL is indicated by higher scores [15]. 

In this study, the overall scale of KIDSCREEN-27 had a Cronbach's alpha coefficient of 0.936. Moreover, all dimensions had satisfactory reliability - physical health (Cronbach's alpha = 0.79), psychological health (Cronbach's alpha = 0.81), autonomy and parents (Cronbach's alpha = 0.85), peers and social support (Cronbach's alpha = 0.87), and school environment (Cronbach's alpha = 0.83). We also calculated the internal consistency of the total questionnaire, which was (Cronbach's alpha = 0.90).

Pilot study

A pilot study of 30 adolescents was conducted to verify that respondents understood the terminology used in the questionnaire. The results of the pilot study are not included in the results section.

Statistical analyses

IBM SPSS Statistics for Windows, version 24 (Armonk, NY: IBM Corp.), was used to analyze the data. We employed descriptive statistics such as mean, standard deviation, and percentage. In addition, a chi-square test was performed to see if there was any association between the categorical variables. A t-test was also used to test some differences between variables. Significant was defined as a probability value of < 0.05.

Ethical consideration

The project was approved by the Standing Committee for Scientific Research of Jazan University (REC-43/05/104). Participants were informed that their participation was voluntary and that the data would be handled confidentially and anonymously. All members of the research team agreed to be held accountable for any scientific or ethical violations.

## Results

Sociodemographic characteristics of the participants

There were a total of 601 responses from the Jazan region in this study. The incomplete response rate was 23.3% (140/601). The questionnaires corresponding to parents who did not consent and adolescents who refused to participate were excluded. The questionnaire was completed by all adolescents who agreed to participate with parental consent, 76.7% (n = 461); 40.1% (185/461) were males and 59.9% (276/461) were females. The average age of the participants was found to be 15.92 ±1.79 years. Regarding the academic level, high school students made up the majority of the participants, 242 (52.4%), with only three illiterate (0.7%). Four hundred forty-three were single (96.1%) and 15 were married (3.3%). Only one divorced individual (0.2%) and two widows (0.4%) were found. Most of the participants lived in villages (63.1%), with the remaining 36.9% in cities (Table 1).

**Table 1:** The sociodemographic characteristics of the participants (n = 461) were split between male and female.

Sociodemographic characteristics	Status	Male N (%), n = 185 (40.1%)	Female N (%), n = 276 (59.9%)	Total N (%) (male + female), n = 461 (100%)	Chi-square P-value
Age	12 year	8 (1.7%)	22 (4.8%)	30 (6.5%)	0.079
	13 year	9 (2.0%)	22 (4.8%)	31 (6.7%)	
	14 year	14 (3.0%)	18 (3.9%)	32 (6.9%)	
	15 year	35 (7.6%)	39 (8.5%)	74 (16.1%)	
	16 year	30 (6.5%)	65 (14.1%)	95 (20.6%)	
	17 year	42 (9.1%)	44 (9.5%)	86 (18.7%)	
	18 year	47 (10.2%)	66 (14.3%)	113 (24.5%)	
Education	Illiterate	2 (0.43%)	1 (0.22%)	3 (0.7%)	0.450
	Elementary school	20 (4.34%)	38 (8.24%)	58 (12.6%)	
	Middle school	69 (15%)	89 (19.31%)	158 (34.3%)	
	High school	94 (20.4%)	148 (32.1%)	242 (52.4%)	
Marital status	Single	176 (38.2%)	267 (57.92%)	443 (96.1%)	0.600
	Married	8 (1.74%)	7 (1.52%)	15 (3.3%)	
	Widow	1 (0.22%)	1 (0.22%)	2 (0.4%)	
	Divorcee	0	1 (0.22%)	1 (0.2%)	
Residence place	Village	137 (29.72%)	154 (33.41%)	291 (63.1%)	0.001
	City	48 (10.41%)	122 (26.5%)	170 (36.9%)	
Residential building	Home	155 (33.62%)	212 (46%)	367 (79.6%)	0.342
	Agency house	2 (0.43%)	4 (0.9%)	6 (1.3%)	
	Rent	22 (4.8%)	48 (10.41%)	70 (15.2%)	
	Others	6 (1.30%)	12 (2.60%)	18 (3.9%)	

Prevalence of bullying 

**Figure 1:**
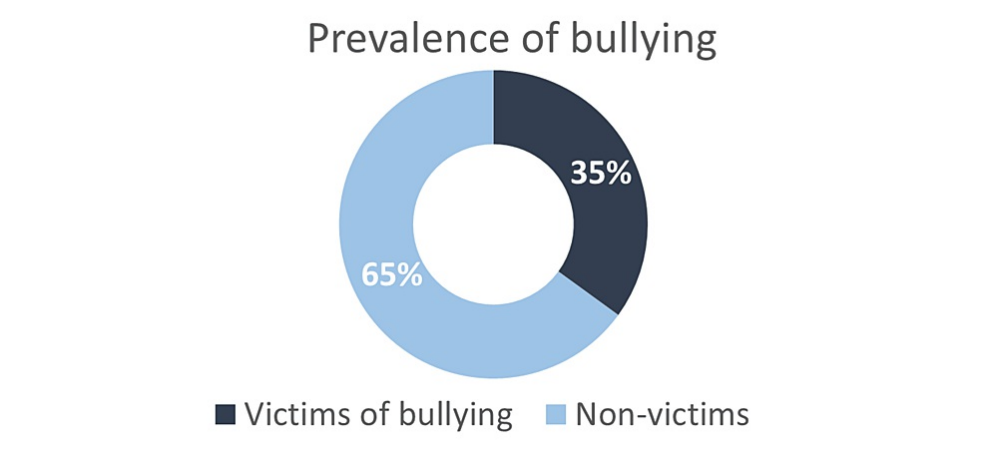
The overall prevalence of bullying in the participants.

Of 461 adolescents enrolled, 163 (35%) reported being bullied compared with 298 (65%) not being bullied (Figure 1). According to the question “how many times have you bullied another person in the previous three months,” the alternative responses were classified into two groups: “victims only” and “bully victims." The first two responses, “never” (52.2%) and “only once or twice” (22.7%), were considered non-bullies, which means that they were “victims only." The alternative responses “two to three times a month” (4.9%), “once a week” (9.8%), and “several times a week” (10.4%) were considered bullies, which means they were both victims and bullies ("bully victim"). According to gender, boys did not significantly tend to bully others more than girls (Table 2).

**Table 2:** Frequency of bullying by others in the last three months by sex.

Gender frequency	Male N (%) n = 82 (17.78%)	Female N (%) n = 81 (17.57%)	Total N (%) (male + female) n = 163 (35.36%)	Chi-square P-value
How many times have you bullied another person in the last three months?				
Never	34 (21%)	51 (31.2%)	85 (52.2%)	0.053
Only once or twice	20 (12.3%)	17 (10.4%)	37 (22.7%)	
Two to three times a month	6 (3.7%)	2 (1.2%)	8 (4.9%)	
Once a week	11 (6.7%)	5 (3.1%)	16 (9.8%)	
Several times a week	11 (6.7%)	6 (3.7%)	17 ( 10.4%)	

Sources and types of bullying by sex

The multiple sources of bullying to which victims are exposed, which are "family," "school," "friends," "strangers," "road," and "internet," are illustrated in Table 3. A total of 56.77% of participants reported being bullied by their friends, which was the most common source of bullying, followed by bullying at school at 53.37%. The internet was used to bully 32.52% of the participants, families were bullied by 24.54%, and 18.40% were bullied by strangers. Lastly, road bullying was the least common, accounting for 13.5%.

**Table 3:** Prevalence, source, and type of bullying in the last three months by sex.

Gender bullying	Male N (%) n = 185 (40.1%)				Female N (%) n = 276 (59.9%)		Total N (%) (male + female) n = 461 (100%)				Chi-square P-value
Have you been bullied?	Yes		No		Yes	No	Yes		No		
Prevalence of bullying	82 (17.78%)		103 (22.34%)		81 (17.57%)	195 (42.3%)	163 (35.36%)		298 (64.64%)		0.001
Source of bullying											
Family	17 (10.42%)			65 (39.88%)	23 (14.11%)	58 (35.58%)	40 (24.54%)		123 (75.46%)		0.256
School	42 (25.77%)			40 (24.53%)	45 (27.60%)	36 (22.09%)	87 (53.37%)		76 (46.63%)		0.579
Friends	53 (32.52%)			29 (17.8%)	41 (25.15%)	40 (24.54%)	94 (57.67%)		69 (42.33%)		0.070
Strangers	13 (7.98%)			69 (42.33%)	17 (10.43%)	64 (39.26%)	30 (18.40%)		133 (81.6%)		0.398
Road	14 (8.59%)			68 (41.72%)	8 (4.91%)	73 (44.79%)	22 (13.5%)		141 (86.5%)		0.179
Internet	19 (11.66%)			63 (38.65%)	34 (20.86%)	47 (28.83%)	53 (32.52%)		110 (67.48%)		0.010
Type of bullying											
Physical		26 (15.95%)		56 (34.36%)	19 (11.66%)	62 (38.03%)	45 (27.6%)	118 (72.4%)		0.239	
Verbal		63 (38.4%)		19 (11.6%)	61 (37.2%)	21 (12.8%)	124 (75.6%)	40 (24.4%)		0.716	
Social		25 (15.34%)		57 (35%)	21 (12.9%)	60 (36.8%)	46 (28.2%)	117 (71.8%)		0.518	
Cyberbullying		16 (9.82%)		66 (40.5%)	30 (18.04%)	51 (31.3%)	46 (28.2%)	117 (71.8%)		0.013	

Table 3 also shows the prevalence of victims who have been exposed to each of the four types of bullying. The most common type was verbal bullying (75.6%), followed by social bullying (28.2%) and cyberbullying (28.2%). The lowest percentage was for physical bullying (27.6%). However, there was no significant association between gender and types/sources of bullying (P > 0.05), except for the internet (P < 0.010) and cyberbullying (P < 0.013). Girls were more bullied than boys.

Prevalence of bullying by academic level

As can be seen in Table 4, bullying prevalence in the last three months by academic level was 0.7% illiterate, 39.5% in elementary school, 33% in middle school, and 35% in high school.

**Table 4:** Being bullied by academic level in the last three months.

Education	Were there bullying situations in the previous three months?		Total
	Yes N (%)	No N (%)	
Illiterate	3 (0.7%)	0	3 (0.7%)
Elementary school	23 (39.5%)	35 (60.5%)	58 (12.6%)
Middle school	52 (33%)	106 (67%)	158 (34.3%)
High school	85 (35%)	157 (65%)	242 (52.4%)
Total	163 (35.36%)	298 (64.64%)	461 (100)

Sources and types of bullying by educational level

**Figure 2:**
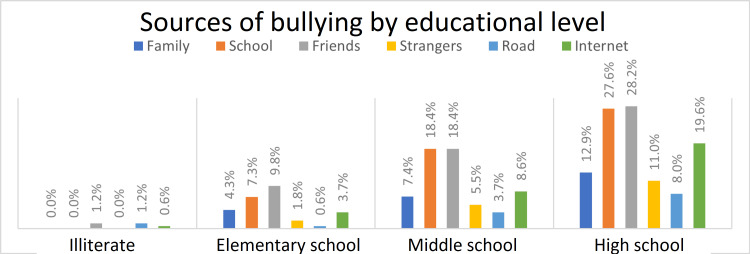
Source of bullying regarding educational level

Figure 2 indicates the sources of bullying in relation to the educational level. Friends were the most common source of bullying in high school, accounting for 28.2%; after that school (27.6%), followed by the internet (19.6%), family (12.9%), strangers (11%), and finally road (8%). The most common sources of bullying in middle school are school and friends equally (18.4%), then the internet (8.6%), then family (7.4%), strangers (5.5%), and finally road (3.7%). In elementary school, the sources with the highest percentages were friends (9.8%), school (7.3%), family (4.3%), the internet (3.7%), strangers (1.8%), and road (0.6%). Last of all, among the illiterates, there is an equal percentage of friends and the road (about 1.2%), then the internet (0.6%).

**Figure 3:**
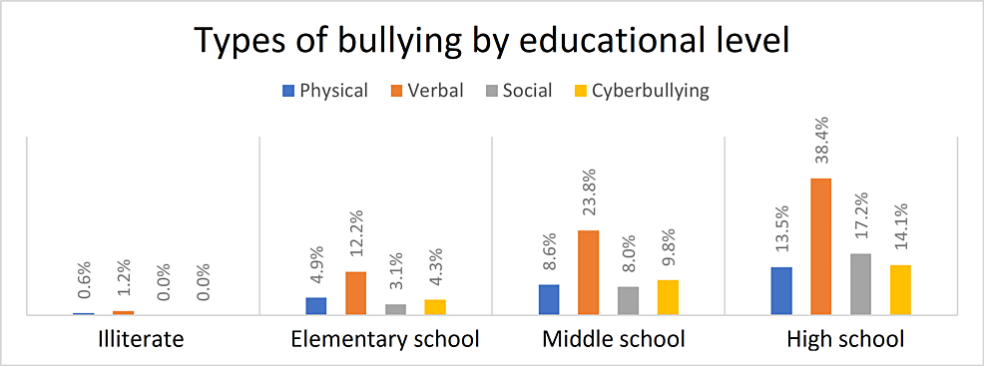
Type of bullying regarding educational level.

In a similar way, the types of bullying by educational level are shown in Figure 3. Illiterates were bullied only verbally (1.2%) and physically (0.6%). Among elementary school students, verbal bullying was the highest (12.2%), then physical (4.9%), cyberbullying (4.3%), and social (3.1%). In middle school, the most common type was also verbal bullying (23.8%), then cyberbullying (9.8%), physical (8.6%), and finally, social bullying (8%). It is also observed that the most common type of bullying in high school was verbal bullying (38.4%), followed by social (17.2%) and cyberbullying (14.1%), while the physical type came in last (13.5%).

Bullying and HRQOL

Table 5 shows the differences between victims and non-victims in the five dimensions of HRQOL measured with KIDSCREEN-27. The findings of the t-test show that there was a highly statistically significant difference between them (P<0.0001). The mean values for the five measures of HRQOL for victims were compared with the mean values of non-victims, and they scored lower than non-victims. Clearly, victims show a poorer overall quality of life (M= 80.5/87.7), as well as in all dimensions - in physical well-being, (M= 80.6/85.2), psychological well-being (M= 78.2/85.6), parent-child relations and autonomy (M= 81.9/89.5), social support and peers (M= 81.9/89), and school (M= 80.8/89.4). The domain which had the lowest mean value among victims was psychological well-being, while parental-child relationships and social support had the highest means.

**Table 5:** Comparison of the M (SD) of scores for victims and non-victims on the five measures of HRQOL. *P<0.05 is statistically significant. M: mean; HRQOL: health-related quality of life

KIDSCREEN_27	Have you been bullied?	N	Mean (±SD)	Minimum	Maximum	P-value
Quality of life	Yes	163	80.5 (±7.8)	60.57	97.60	0.000*
	No	298	87.7 (±6.6)	63.59	99.21	
Physical well-being	Yes	163	80.6 (±9.7)	60	100	0.000*
	No	298	85.2 (±8.7)	60	100	
Psychological well-being	Yes	163	78.2 (±8.4)	60	100	0.000*
	No	298	85.6 (±7.6)	64	100	
Parent relations and autonomy	Yes	163	81.9 (±9.8)	60	100	0.000*
	No	298	89.5 (±8.4)	60	100	
Social support and peers	Yes	163	81.9 (±11)	60	100	0.000*
	No	298	89 (±10)	60	100	
School	Yes	163	80.8 (±10)	60	100	0.000*
	No	298	89.4 (±8.9)	60	100	

## Discussion

Our study revealed that 35% of the adolescents had been bullied within the previous three months. This finding is supported by a systematic review, which indicated that the average prevalence of bullying is 35%, with victimization occurring in 36% of individuals of 12-18 years of age [16]. Furthermore, those who were bullies and victims ("bully victims") represented almost a quarter of all victims. In line with the literature, a significant relationship was found between gender and victimization, with boys suffering from bullying at much higher rates than girls [17,18]. We also observed that boys' victims, "bully victims," showed no significantly increased tendency to bully others.

A breakdown of the results shows that bullying was more common among elementary students than among middle and high school students. This finding is consistent with a previous study finding conducted among young people in the United States [19].

Although previous research has focused on types of bullying, in this study, the collected data contributed to a better understanding of where bullying comes from. Therefore, it is worth noting that bullying can occur in a variety of situations and relationships, not just at school. In general, friends of the participants were the most prevalent source of bullying, followed by bullying at school, on the internet, from family, strangers, and, lastly, from the road. Data also suggest that most of the participants were verbally bullied, followed by social and cyberbullying, which were at the same levels, and lastly, physical bullying. The findings of this study were supported by the claims of previous research that revealed that verbal bullying represents 70.0% of all reported cases, and can have both short-and long-term consequences for the individual [20]. From Coloroso's perspective, words have a strong impact on a child’s soul that can continue into adulthood, as they may not heal from what they experienced at school [21]. Additionally, these different types and sources of bullying vary by gender and educational level of victims. According to our findings, boys were more likely to report verbal and physical bullying and were typically bullied by their friends, whereas stranger bullying seems to be the least common. On the contrary, girls were more likely to report verbal and cyberbullying, as well as more likely to be bullied at school. The results showed that road bullying was not practiced frequently among girls. Regarding sex differences, boys were slightly more likely than girls to be physically abused, while girls were significantly more likely than boys to be targeted by cyberbullying. These findings are supported by previous work [22,23]. However, there are no gender differences in terms of other sources and types of bullying. According to Smith et al., gender, in conjunction with cultural norms, makes girls more prone to online platforms linked with relational interactions (e.g., social media), whereas boys may be more susceptible to types of bullying in which differences and independence may be shown through direct violence and fighting [24].

From our point of view, another likely explanation of why girls suffer from cyberbullying more than boys may be that girls usually have fewer relationships in real life and generally spend more time on social media. In addition, girls are more likely to engage in indirect types of bullying, such as spreading rumors or pretending to be someone else, which is easier to do on the internet [17]. Another possible explanation is that in our culture, the stereotype of girls is more conservative than that of boys. Therefore, any behavior opposite to this, or any socially unacceptable act on social media, will expose them to bullying.

The second point to consider is the educational level. According to the data, students in elementary school were by far the most susceptible to being bullied physically and verbally and were mainly targeted by their friends. Verbal and cyberbullying were the most common forms of bullying among middle school students, who were tormented by their friends and school colleagues. Participants were more likely to experience verbal and social bullying among high schoolers and were typically bullied by their friends. As for the illiterates, they were subjected to verbal bullying not only by their friends but also by people on the road. Most studies agree that older adolescents tend to be bullied verbally and socially [25], but younger adolescents seem to be more exposed to physical bullying [26]. This is attributed to the fact that older adolescents have mastered all the necessary linguistic and social skills for these types of violence.

On the other hand, our hypothesis had been verified, as expected. The analysis clearly supports the theory that bullying is negatively associated with HRQOL. A previous study found this association [1]. Our results indicate that victims of bullying have lower HRQOL compared to nonvictims in all dimensions of KIDSCREEN-27. They were found to have poorer physical well-being, poorer psychological well-being, reduced feelings of autonomy and relationships with parents, reduced feelings about peers and social support, and reduced feelings about the school environment. It is not surprising that "psychological well-being" was the domain most affected, which addresses positive emotions and happiness in life, as well as the absence of negative emotions such as isolation and sadness. However, parent/child relationships and autonomy, although they had lower scores in victims, had slightly higher ratings compared to other domains. It may explain the low prevalence rates of bullying in families compared to others. Hence, the results of this study can be used to design health education programs that address this important issue and improve the HRQOL of adolescents.

Limitations of the study

There are two important limitations to this study that should be highlighted and discussed. Since the study is cross-sectional, the causative relationship cannot be established. Thus, further investigations are needed to confirm these findings; longitudinal studies could examine this relationship in more depth and verify the direction of the association observed here. Beyond that, the distribution was not equal across educational levels due to the low response rate, particularly among illiterate and elementary school students, which may not reflect their actual victimization. Therefore, it is proposed to increase the number of participants and use direct data collection methods such as hand-to-hand surveys.

## Conclusions

Based on our results, it is reasonable to conclude that bullying is high among adolescents in the Jazan region, especially verbal bullying and being bullied by their friends. Boys suffered significantly more from bullying than girls. However, cyberbullying victimization was significantly associated with girls. Beyond that, we found that being bullied was strongly associated with lower HRQOL in almost all aspects of daily activities. In light of the high prevalence of bullying, we urge families to pay more attention to the psychological difficulties of their adolescent children and encourage collaboration among professionals in the sectors of education, health, and social services to understand the unique problems that adolescents experience and identify bullying. To expand the knowledge about bullying and effective intervention, further research is needed on high-risk indicators.
